# The Chemotherapeutic Drug 5-Fluorouracil Promotes PKR-Mediated Apoptosis in a p53- Independent Manner in Colon and Breast Cancer Cells

**DOI:** 10.1371/journal.pone.0023887

**Published:** 2011-08-24

**Authors:** María Angel García, Esther Carrasco, Margarita Aguilera, Pablo Alvarez, Carmen Rivas, Joaquin María Campos, Jose Carlos Prados, Miguel Angel Calleja, Mariano Esteban, Juan Antonio Marchal, Antonia Aránega

**Affiliations:** 1 Unidad de Investigación, Hospital Universitario Virgen de las Nieves, Granada, Spain; 2 Instituto de Biopatología y Medicina Regenerativa (IBIMER), Universidad de Granada, Granada, Spain; 3 Unidad de Farmacogenética, Hospital Universitario Virgen de las Nieves, Granada, Spain; 4 Centro Nacional de Biotecnología, CSIC, Madrid, Spain; 5 Departamento de Química Farmacéutica y Orgánica, Facultad de Farmacia, Universidad de Granada, Granada, Spain; University of Chicago, United States of America

## Abstract

The chemotherapeutic drug 5-FU is widely used in the treatment of a range of cancers, but resistance to the drug remains a major clinical problem. Since defects in the mediators of apoptosis may account for chemo-resistance, the identification of new targets involved in 5-FU-induced apoptosis is of main clinical interest. We have identified the ds-RNA-dependent protein kinase (PKR) as a key molecular target of 5-FU involved in apoptosis induction in human colon and breast cancer cell lines. PKR distribution and activation, apoptosis induction and cytotoxic effects were analyzed during 5-FU and 5-FU/IFNα treatment in several colon and breast cancer cell lines with different p53 status. PKR protein was activated by 5-FU treatment in a p53-independent manner, inducing phosphorylation of the protein synthesis translation initiation factor eIF-2α and cell death by apoptosis. Furthermore, PKR interference promoted a decreased response to 5-FU treatment and those cells were not affected by the synergistic antitumor activity of 5-FU/IFNα combination. These results, taken together, provide evidence that PKR is a key molecular target of 5-FU with potential relevance in the clinical use of this drug.

## Introduction

The fluoropyrimidine 5-fluorouracil (5-FU) is widely used in the treatment of a range of cancers including colorectal cancer and breast tumors [1], [2], but resistance to the drug remains a major clinical problem. The antimetabolite 5-FU is an analogue of uracil with a fluorine atom at the C5 position of the pyrimidine ring. Inside cells, 5-FU is converted into different active metabolites, including fluorodeoxyuridine monophosphate (FdUMP), fluorodeoxyuridine triphosphate (FdUTP), and fluorouridine triphosphate (FUTP). These metabolites have been implicated in both global RNA metabolism due to the incorporation of the ribonucleotide FUMP into RNA, and DNA metabolism due to thymidylate synthase (TS) inhibition or direct incorporation of FdUMP into DNA, leading to a wide range of biological effects which can act as triggers for apoptotic cell death [3], [4]. The tumor suppressor p53 is one molecular target by 5-FU, and as part of its antitumor activity triggers cell cycle blockage and/or apoptosis. However, while several reports have demonstrated that 5-FU-induced apoptosis is dependent on the tumour suppressor p53 protein, apoptosis can also occur in mutant p53 cell lines by a mechanism still unknown [5], [6], [7].Since alterations in the mediators of 5-FU-induced apoptosis may account for chemo resistance, the identification of new targets involved in the 5-FU-induced apoptosis is of significant clinical interest.

Interferons (IFN) type I are pleiotropic cytokines with antiviral and antitumor activities that are widely used in the clinic [8], [9]. In clinical trials and experimental settings the combination of IFNα with 5-FU has been demonstrated to be superior to 5-FU administered alone, although the mechanism of this synergistic effect is still poorly understood [10], [11], [12], [13]. Approximately 35 years ago the double-stranded RNA (dsRNA)-dependent protein kinase (PKR) was initially identified as an innate immune anti-viral protein that is induced by the IFN type I [14], [15]. Since then, PKR has been linked to normal cell growth and differentiation, inflammation, cytokine signaling, and apoptosis, and is involved in the antiviral and antitumor activities of IFN [16]. Moreover, altered PKR activity has been shown to play a role in neurodegenerative diseases and cancer [17], [18], [19], [20]. PKR is a serine/threonine kinase, characterized by two distinct kinase activities: autophosphorylation, which represents the activation reaction, and the phosphorylation of the translation initiation factor eIF2α [21], which impairs eIF-2 activity, resulting in the inhibition of protein synthesis [22]. In addition, PKR has a role in signal transduction and transcriptional control through the IkB/NF-kB pathway [23], [24]. PKR is activated in response to dsRNA of cellular, viral, or synthetic origin (poly IC). PKR can also be activated by polyanions such as heparin, dextran sulfate, chondroitin sulfate, and poly-L-glutamine [25]. Cellular stress such as arsenite, thapsigargin, H_2_O_2_, ethanol and ceramide can also activate PKR, [26], [27] presumably through the PKR-associated activator PACT/RAX protein [28]. Over-expression or continued activation of PKR leads to cell death by apoptosis through the FADD/caspase 8 and mitochondrial APAF/caspase 9 activation pathways [29], [30], [31].

The aim of this work was to analyze the role of PKR as a molecular target of 5-FU and the importance of PKR in the enhanced antitumor activity induced by 5-FU and IFNα treatment. We have identified PKR as a kinase responsible for 5-FU-induced phosphorylation of the translation initiation factor eIF2α, and of apoptosis induction, in a p53 independent manner in colon and breast tumor cells. In addition, we show that cells with PKR knockdown respond poorly to 5-FU treatment and are not susceptible to the synergistic antitumor activity produced by the 5-FU/IFNα combination.

## Materials and Methods

### Cell culture and reagents

PKR^+/+^ and PKR^−/−^ MEFs [32], and human colon HCT116 p53^+/+^ and p53^−/−^ cells [7], were kindly provided by M Esteban (National Center of Biotechnology, Spain) and B. Vogelstein (Johns Hopkins Oncology Center, USA), respectively. EIF2α^S/S^ and eIF2α^A/A^ MEFs were kindly provided by C De Haro (Centro de Biología Molecular Severo Ochoa, Spain) [33]. Human colon SW-480, T84, RKO, cell lines and the human breast MCF7, MDA-MB-231, T47D cell lines were obtained from American Type Culture Collection (ATCC). PKR interference with short hairpin RNAs targeting PKR (shRNA-PKR) were prepared as described below. Most of the cells were maintained in DMEM medium supplemented with 10% FBS, 1% penicillin-streptomycin and 1% non-essential amino acid solution. MDA-MB-231 cell line was cultured in DMEM/F12-Ham medium. Exponentially growing cells were used for all experiments. 5-FU (Sigma-Aldrich, F6627-1G) was dissolved in DMSO and stored at −20°C. For each experiment, the stock solutions were further diluted in medium to obtain the desired concentrations. IFNalpha 2b (Intron A) was obtained from Schering-Plough.

### Cell survival assay

The effect of 5-FU on cell viability was assessed using the sulforhodamine-B colorimetric assay (SRB). Aliquots of cell suspension (3×10^3^ cells/well) were seeded onto 24-well plates and incubated for 24 h. The cells were then treated with different concentrations of 5-FU and with or without IFNα (50 IU/ml) in the culture medium. Three days later, the wells were aspirated, fresh medium and treatment were added, and cells were maintained for three additional days. Thereafter, cells were processed as described previously [34], using a Titertek Multiscan apparatus (Flow, Irvine, CA, USA) at 492 nm. We evaluated linearity of the SRB assay with cell number for each cell stock before each cell growth experiment. The IC_50_ values were calculated from semi-logarithmic dose–response curves by linear interpolation. All experiments were plated in triplicate wells and were carried out at least twice.

### Apoptosis analysis

Cells were plated in 6-well plates and maintained in the incubator overnight. Cells were then induced for 48 hours with 5-FU alone or in combination with IFNα (500 IU/ml). IFNα was added 16 hours before the 5-FU.After 72 hours, cells were trypsinized and analyzed using the kit TACS™ Annexin V-FITC (R&D System, TA4638), and the Annexin V-APC Apoptosis detection kit (ebioscence, 88-8007-74) for cells expressing shRNA-PKR. The samples were immediately processed with a FACScan flow cytometer “VANTAGE” (Becton Dickson, San Jose, CA, USA), using the flow cytometry service of the Scientific Instrumental Center at the University of Granada.

### Cell cycle analysis

Cells in exponential growth were plated on 6-well plates and maintained in the incubator overnight. After that, cells were induced for 24 and 48 hours with 10 µM 5-FU. Cells were harvested, washed twice with PBS and fixed in 70% (vol/vol) cold ethanol for up to 1 week. Cells were centrifuged, the pellet washed once with PBS and re-suspended in 250 µl of Propidium Iodide (PI) solution (100 µl/ml RNAsa, 40 µl/ml PI in PBS) for 30 min in the dark at 37°C. The samples were immediately analyzed using a FACScan flow cytometer (Becton Dickinson, San Jose, CA, USA).

### Western Blot analysis

Cells were plated on 6-well plates in their respective medium. After treatment, medium was removed and the cells were lysed in Laemmli buffer. The protein sample was subjected to electrophoresis, transferred onto nitrocellulose membranes (Bio-rad,162-0115), and blocked in PBS containing 5% non-fat dry milk for 1 h at room temperature. Primary antibodies used included a polyclonal antibody to total human PKR provided by M Esteban (National Center of Biotechnology, Spain), a polyclonal to phospho- PKR (Thr 451) (Calbiochem, 527460), a polyclonal antibody to phospho-eIF2α (Ser 51) (Invitrogen, 44–728,G), a polyclonal antibody to phospho-p53 (Ser 15)(Cell signaling, 92845), a polyclonal antibody to full and cleaved caspase 3 (Cell signaling, 8G10) and a monoclonal antibody to β-actin (Sigma, A2228). Secondary antibodies used included anti-rabbit IgG peroxidase conjugate (Sigma, A0545) and anti-mouse IgG peroxidase conjugate (Sigma, A9044). Bands were visualized using the ECL system (Amersham Pharmacia Biotech, UK).

### Immunofluorescence

HCT116 p53^+/+^ and p53^−/−^ cells were mock-treated or treated with 5 µM of 5-FU. At indicated times, cells were washed with phosphate-buffered saline (PBS), fixed with 4% paraformaldehyde, and permeabilized (10 min, room temperature) with 0.1% Triton X-100 in PBS, washed, and blocked with 20% bovine serum albumin in PBS. Cells were incubated (2 h, room temperature) with anti Phospho-PKR (Thr 451, Sigma) or anti- PKR antibody. Cover slips were washed extensively with PBS and were further incubated (1 h, 37°C) with DAPI and appropriate fluorescein-conjugated isotype-specific secondary antibodies (Santa-Cruz). Images were obtained using a Bio-Rad Radiance 2100 confocal laser microscope.

### Knocking down expression of human PKR in colon and breast cancer cell lines by shRNA

To knockdown PKR, we obtained pGIPZ-lentiviral shRNAmir vectors containing either non-silencing (NS) scrambled sequence or three hairpin sequences targeting PKR (Open Biosystems, RHS4430). The pGIPZ vectors expressed GFP to identify transfected cells. MCF7, and HCT116 p53^+/+^ and p53^−/−^ cells were transfected using Amaxa Cell line nucleofector kit V (Lonza Cologne AG, VCA-1003) according to the manufacturers protocol. To generate stable cell lines, two days after transfection, we added puromycin (Invivogen, 58-58-2) at a concentration of 1 µg/ml to HCT116 cells and 3 µg/ml to MCF-7 cells to select puromycin-resistant clones.

### RNA extraction and Real-Time Quantitative RT-PCR Analysis

RNA direct isolation from tumor cell lines was performed by RNeasy mini kit (Qiagen 74106), and sequentially complementary DNA was generated using reverse transcription system (Promega A3500), both strictly following the manufacturing instructions. Real-time-PCR Quantitative was performed in 96-well plates with an Applied Biosystems 7500 Real Time PCR System using TaqMan Gene Expression Assays (Applied Biosystems) to quantify the amplicons. Assays include PKR (Hs00169345_ml) and GAPDH (Hs 99999905_ml) that was used as endogenous control to normalize RNA concentration. PCR reaction was performed following a protocol standardized by Applied Biosystems.

## Results

### PKR protein is activated by 5-FU

**Figure 1:**
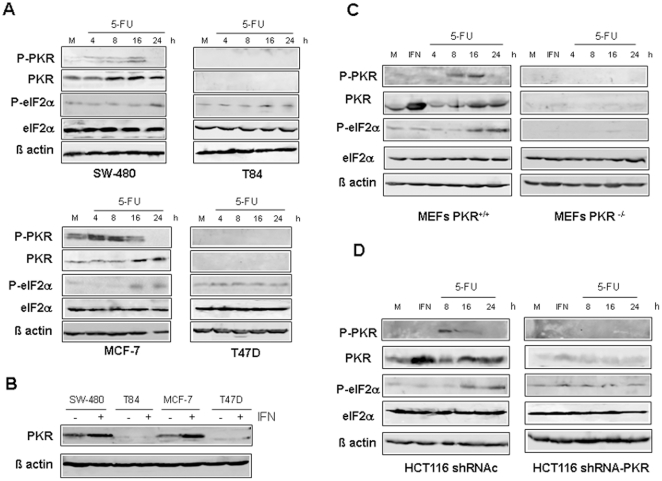
PKR activation upon 5-FU treatment in mouse and human cell lines. (**A**) SW-480, T84, MCF-7 and T47D cell lines were mock-treated or treated with 10 µM of 5-FU during 4, 8, 16 and 24 hours. (**B**) SW-480, T84, MCF-7 and T47D cell lines were mock-treated or treated with 500 IU/ml of IFNα during 16 hours. (**C**) PKR^+/+^ and PKR^−/−^ MEFs and (**D**) HCT-116 colon cell line expressing shRNA against PKR (shRNA-PKR) or expressing a control shRNA (shRNAc) as have been described in the Material and Methods, were mock-treated, treated with 500 IU/ml of IFNα during 16 hours or treated with 10 µM of 5-FU during 4, 8, 16 and 24 hours. Total proteins were extracted for immunoblot analysis using anti-phospho PKR, anti- whole PKR, anti-phospho eIF2α, anti-whole eIF2α and anti-β-actin antibodies.

In order to explore if PKR is activated in response to 5-FU, we first analyzed PKR phosphorylation and that of its natural substrate eIF2α in several human colon and breast cancer cell lines. 5-FU treatment induced PKR phosphorylation in colon cancer SW-480 cells by 4 hours with a peak at 16 hours, whereas in breast cancer MCF7 cells, PKR activation was already detected 4 hours after treatment. The translation initiation factor eIF2α was phosphorylated in both cell lines in response to 5-FU (Figure 1A). Importantly, we also observed an increase in the PKR levels after 5-FU treatment. In contrast, we found one colon (T84) and one breast cell line (T47D) where the PKR protein was not detected and the basal level of eIF2α phosphorylation was maintained during the 5-FU treatment. Moreover, IFNα treatment upregulated PKR level in the tumor cell lines analyzed except in T84 and T47D cells (Figure 1B). To confirm the implication of PKR in eIF2α phosphorylation by 5-FU, we treated mouse embryonic fibroblasts (MEFs) derived from wild type (PKR^+/+^) or PKR knockout (PKR^−/−^) mice with the chemotherapeutic agent. As shown in Figure 1C, 5-FU treatment induced the upregulation and activation of the PKR protein as well as phosphorylation of eIF2α, whereas in PKR^−/−^-drug treated cells, eIF2α phosphorylation was not detected. Moreover, in human HCT116 colon cancer cell line in which PKR was knocked down by RNA interference using shRNA-PKR, treatment with 5-FU did not increase the basal level of eIF2α phosphorylation, while eIF2α was phosphorylated in cells expressing a control shRNA (Figure 1D). These data indicate that PKR is involved in the biological mechanism exerted by 5-FU.

### PKR plays an important role in the apoptosis induced by 5-FU

**Figure 2:**
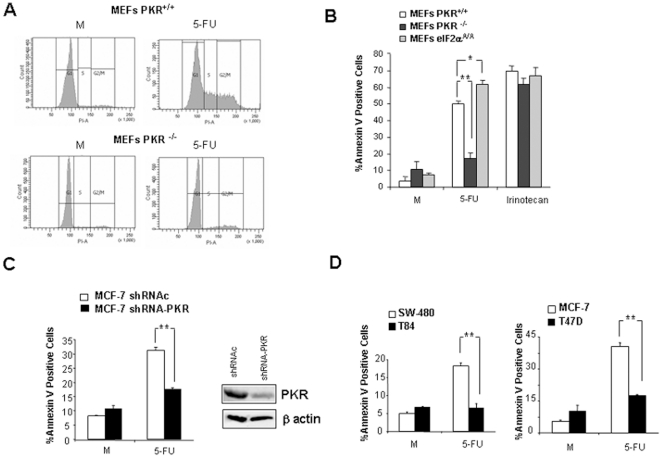
PKR implication in the cell cycle arrest and in the apoptosis induced by 5-FU. (**A**) PKR^+/+^ and PKR^−/−^ MEFs were mock treated or treated with 5 µM of 5-FU during 48 hours. Cell cycle was analyzed by flow cytometry after staining with PI. (**B**) PKR^+/+^ MEFs, PKR^−/−^ MEFs and eIF2α^A/A^ MEFs were mock-treated, treated with 10 µM of 5-FU or with 100 µg/ml of Irinotecan during 48 hours. Thereafter treated cells were trypsinized and analyzed by flow cytometry to AnnexinV positive determination. The asterisks ‘‘**” designates p<0.01 and the asterisk ‘‘*” designates p<0.05 in mutated cells versus wild type cells. (**C**) MCF-7 breast cell line expressing shRNA against PKR (shRNA-PKR) or expressing a control shRNA (shRNAc) and (**D**) colon and breast cancer cell lines SW-480, T84, MCF-7 and T47D, were mock-treated, or treated with 10 µM of 5-FU during 48 hours. Subsequently, cells were trypsinized and analyzed by flow cytometry to AnnexinV positive determination. Values shown represent the mean of the triplicate determinations calculated from a single experiment. Experiments were repeated at least three times. The asterisks ‘‘**” designates p<0.01 in T84 versus SW-480 cells and T47D versus MCF-7 cells.

To demonstrate the role of PKR in cell cycle arrest and apoptosis induced by 5-FU, PKR^+/+^ and PKR^−/−^ MEFs were treated with 5-FU during 48 hours and analyzed by flow cytometry. As shown in Figure 2A, 5-FU induces an accumulation of both S and G2/M phases (13.2% and 27.8%, respectively) in comparison with non-treated cells (3.1% in S phase and 7% in G2/M phase) in PKR^+/+^ MEFs. However, 5-FU treatment induced in PKR^−/−^ MEFs, a weak accumulation in both S and G2/M phases (3.9% and 10.6%, respectively) in comparison with non-treated cells (2.4% in S phase and 4.2% in G2/M phase). Moreover, treatment with 10 µM of 5-FU in PKR^+/+^ MEFs induced considerable levels of apoptosis (Figure 2B). However, apoptosis was not induced in 5-FU-treated PKR^−/−^ cells. To explore the role of eIF2α phosphorylation during 5-FU treatment, we also analyzed MEFs expressing non phosphorylatable eIF2α bearing the S51A mutation (i.e., eIF2α^A/A^ MEFs). We observed that eIF2α^A/A^ MEFs were more susceptible to cell death by 5-FU than PKR^+/+^ MEFs (Figure 2B). Irinotecan-treatment was used as a positive control of apoptosis induction [35].

To determine if in human cells PKR is also required for the induction of apoptosis in response to 5-FU treatment, PKR was down-regulated using shRNA against PKR in breast MCF-7 cancer cells and then, effectiveness of chemotherapeutic treatment was analyzed and compared to that observed after the treatment of the same cells expressing control dsRNA (Figure 2C). In fact, apoptosis induced by 5-FU was significantly reduced in those cells transfected with shRNA against PKR. It is interesting to note that the colon cancer T84 cell line showed no apoptosis induction when it was compared with the SW-480 colon cancer cell line in response to 10 µM of 5-FU, and the breast cancer T47D cell line showed a lower level of apoptosis compared with the MCF-7 breast cancer cell line, also treated with 10 µM of -FU (Figure 2D). The results of Figure 2 showed the importance of PKR activation in the apoptosis induced by 5-FU treatment.

### PKR is involved in the cytotoxic effect of 5-FU and in the effectiveness of the 5-FU/IFNα-combined therapy

**Figure 3:**
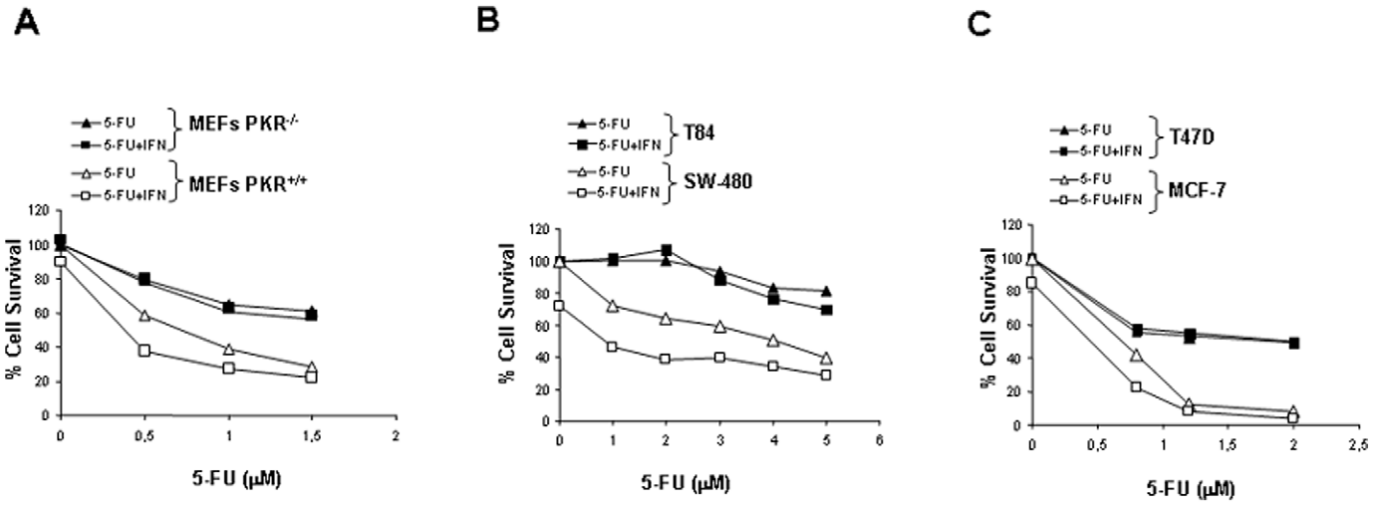
Cytotoxicity effect of 5-FU- and 5-FU/IFNα-combined therapy. (**A**) PKR^−/−^ and PKR^+/+^ MEFs, (**B**) Human colon cancer cell lines T84 and SW-480, and (**C**) Human breast cancer cell lines, T47D and MCF-7 were treated with increasing amounts of 5-FU alone (triangles) or in combination with 50 IU/ml of IFNα (square) during 6 days as described in Material and Methods. The curve of cell survival was represented as percentage referred to mock-treated cells. The concentration of 5-FU was different depending on cell lines sensitivity. Values shown represent the mean of the triplicate determinations calculated from a single experiment. Cell survival was statistically different when comparing cell lines expressing PKR with the cell lines deficient in PKR expression (p≤0.05 in A and B; p≤0.025 in C). Experiments were repeated at least three times.

Taking into account the role of PKR in the cellular response to 5-FU, we decided to analyze the 5-FU cytotoxic effect in cells lacking or expressing PKR. Moreover, we hypothesized that the up-regulation of the PKR level produced by IFNα could be involved in the well known enhanced antitumor activity of this chemotherapeutic drug [12]. To prove this assumption, cell survival was determined in PKR^+/+^ and PKR^−/−^ MEFs treated with increasing amounts of 5-FU, either alone or in combination with 50 IU/ml of mouse IFNα. Our results showed that the cytotoxic effect of 5-FU was higher in PKR^+/+^ MEFs in comparison with the PKR^−/−^ MEFs (Figure 3A). Moreover, mouse IFNα improved the cytotoxic effect of 5-FU only in PKR^+/+^ MEFs. The 5-FU concentration necessary to produce 50% cell death (IC_50_ values) is shown in Table 1. Similar experiments were carried out in human colon and breast cancer cell lines (Figure 3B and C). The cytotoxic effect of 5-FU was higher in those cell lines expressing PKR protein in comparison with cells where PKR was not detected. In fact, the 5-FU IC_50_ value was about 2-fold higher in T84 cells than in SW-480 cells (Table 1). Moreover, the 5-FU IC_50_ value in T47D cells was about 4-fold higher than in MCF7 cells (Table 1). Therefore, cells lacking PKR like T84 and T47D, were not affected by the addition of human IFNα to the 5-FU treatment. In contrast, IFNα increased the cytotoxic effect of 5-FU and reduced the 5-FU IC_50_ in SW-480 and MCF7 cells (Figure 3B and C; Table 1).

**Table 1:** IC_50_ values derived from curves analysis showed in Fig. 3.

Cell Lines	IC50(µM)	
	5-FU	5-FU+IFNα
MEFs PKR^+/+^	0.88±0.06	0.61±0.02
MEFs PKR^−/−^	1.74±0.05	1.74±0.22
SW-480	3.70±0.09	1.90±0.20
T84	7.48±0.60	7.80±0.10
MCF-7	0.71±0.04	0.52±0.08
T47D	2.80±0.10	2.78±0.23

**Figure 4:**
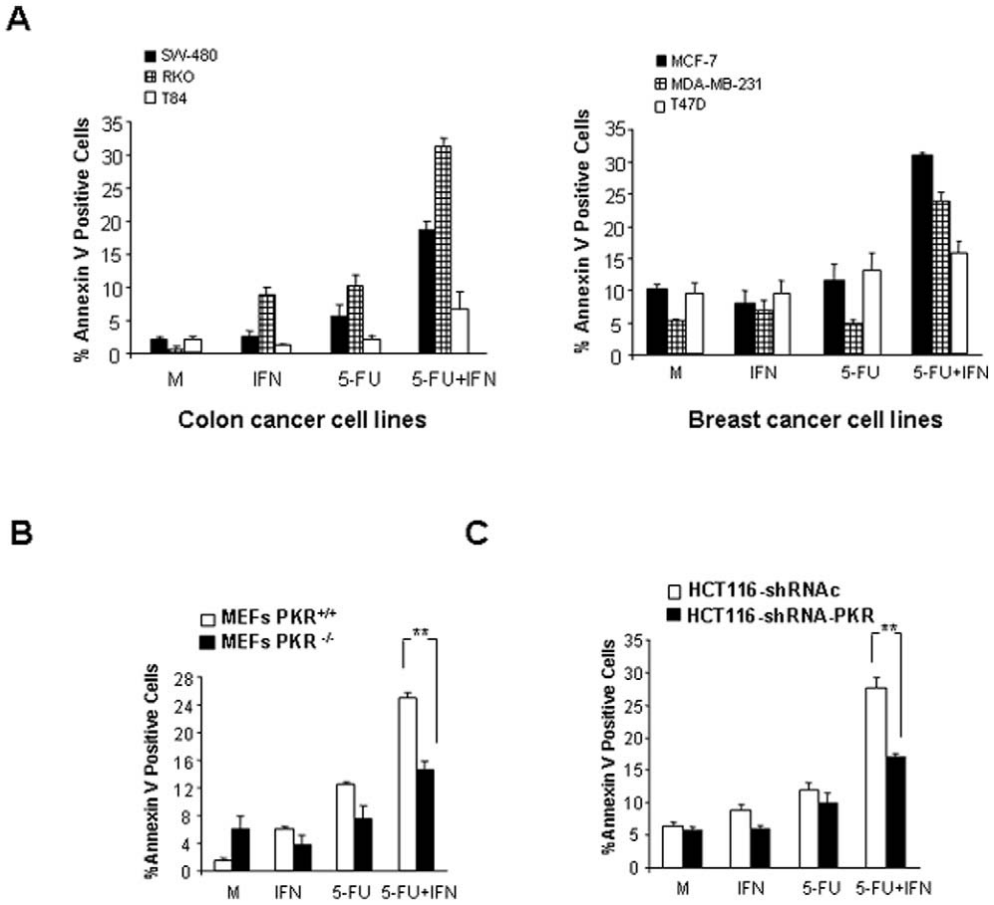
Apoptosis induced in MEFs and in human colon and breast cancer cell lines during the 5-FU/IFNα-combined treatment. (**A**) 5×10^5^ human colon and breast cancer cells, (**B**) 5×10^5^ PKR^+/+^ or PKR^−/−^ MEFs and (**C**) 5×10^5^ HCT-116 colon cancer cells expressing shRNA against PKR or control shRNA, were mock-treated, treated with 500 IU/ml of IFNα, treated with 5 µM of 5-FU or treated with combination of IFNα plus 5-FU during 48 hours. Thereafter treated cells were trypsinized and analyzed by flow cytometry to Annexin V positive determination. The asterisks “**” designates p<0.01 and the asterisk “*” designates p<0.05 in cells with PKR knockdown versus cells expressing PKR.

To confirm that through the induction of apoptosis PKR activation is involved in the synergistic effect exerted by 5-FU/IFNα combined treatment, we first set up the conditions by which either, 5-FU or IFNα (i.e, 5 µM of 5-FU and 500 IU/ml of IFNα) did not induce or induce low levels of apoptosis under our experimental conditions in the different tumor cell lines (Figure 4). When 5-FU and IFNα were combined, they were able to induce a considerable level of apoptosis in colon and breast cancer cell lines expressing the PKR protein. In contrast, those cells lacking PKR (T84 and T47D cell lines) were not susceptible to the 5-FU/IFNα induced apoptosis (Figure 4A). We also analyzed PKR^+/+^ and PKR^−/−^ MEFs, and HCT116 cells expressing the shRNA control or the shRNA against PKR. As shown in Fig. 4B, the apoptosis induced by the combined 5-FU/IFNα treatment was significantly reduced in PKR^−/−^ MEFs compared with PKR^+/+^ MEFs. Interference of PKR in HCT116 cells also triggered a significant reduction in apoptosis levels compared with the cells expressing the shRNA control (Figure 4C). These results implicate PKR in the cytotoxic effect of 5-FU and suggest that IFNα enhances this effect, in part, through PKR up-regulation.

### PKR activation by 5-FU occurs independently of p53

**Figure 5:**
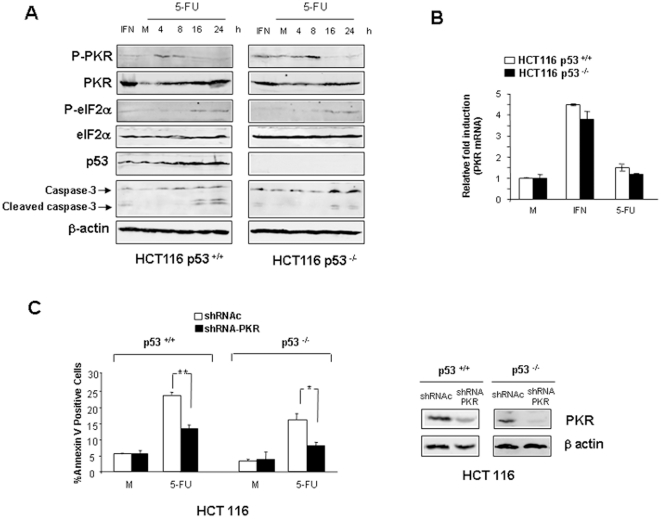
PKR regulation by 5-FU treatment in HCT116 p53^+/+^ and HCT116 p53^−/−^ cells. (**A**) HCT116 p53^+/+^, HCT116 p53^−/−^ cells were mock-treated, treated with 500 IU/ml of human IFNα during 16 hours, or treated with 10 µM of 5-FU during 4, 8, 16 and 24 hours. Total proteins were extracted for immunoblot analysis using anti whole p53, PKR, anti phospho PKR, anti whole PKR, anti phospho eIF2α, anti whole eIF2α, anti cleaved caspase-3, and anti β-actin antibodies. (**B**) HCT116 p53^+/+^, HCT116 p53^−/−^ cells were mock treated or treated with 10 µM of 5-FU or 500 IU/ml of IFNα during 16 hours. Expression of endogenous mRNA of PKR was analyzed by real-time RT-PCR (means±SEM, n = 3) as described in material and methods. (**C**) HCT116 p53^+/+^, HCT116 p53^−/−^ cells expressing shRNA against PKR or control shRNA mock-treated, or treated with 10 µM of 5-FU during 48 hours. After treatment cells were trypsinized and analyzed by flow cytometry to AnnexinV positive determination. Total proteins of both cell lines were extracted for immunoblot analysis using anti whole PKR and β-actin antibodies. The asterisks “**” designates p<0.01 in HCT116 p53^+/+^ cells expressing shRNA against PKR versus cells expressing the control shRNA. The asterisk “*” designates p<0.05 in HCT116 p53^−/−^ cells expressing shRNA against PKR versus cells expressing the control shRNA.

**Figure 6:**
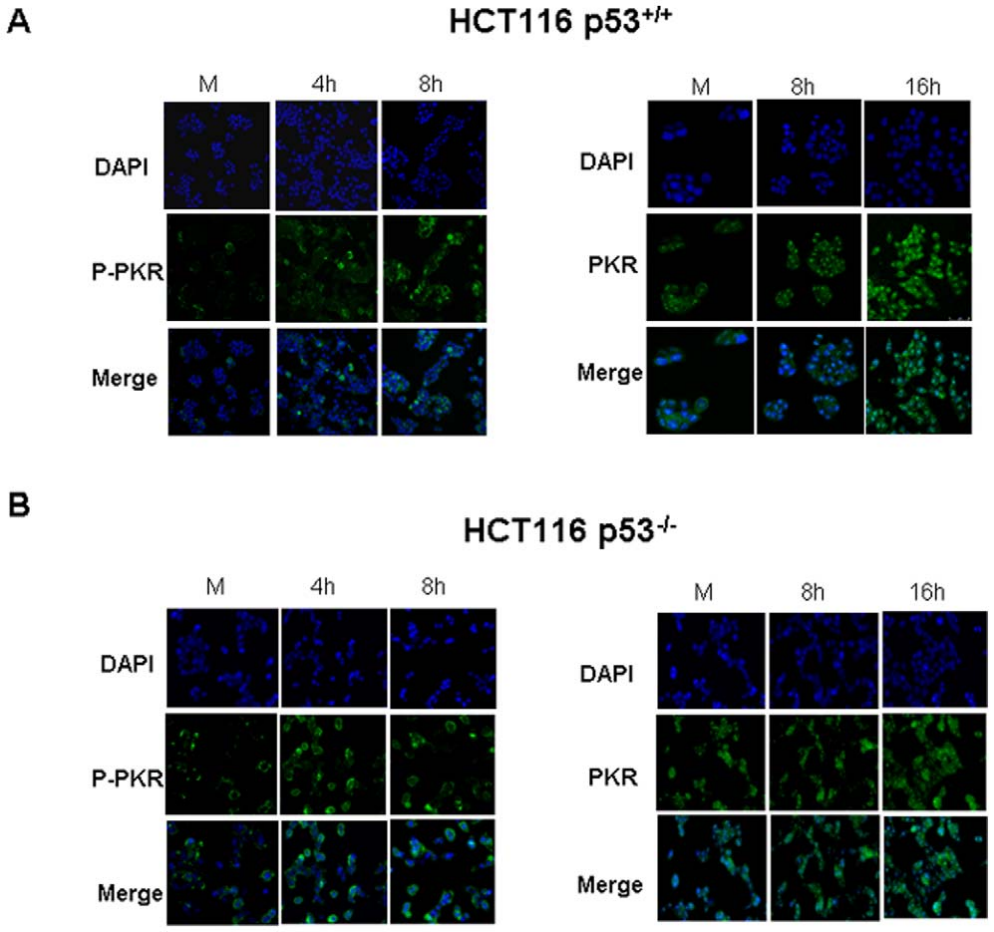
Localization of phospho-PKR and total-PKR in HCT116 p53^+/+^ and HCT116 p53^−/−^ cells. (**A**) HCT116 p53^+/+^ cells and (**B**) HCT116 p53^−/−^ cells were mock treated or treated with 5 µM of 5-FU. At the indicated times cells were fixed and processed for immunofluorescence analysis by confocal microscopy as described in Material and Methods using antibodies against phospho-PKR (green) and anti- whole PKR (green) and DAPI for staining nuclei (blue).

It has been previously described that the induction of apoptosis in response to 5-FU was mediated by p53 [36]. Moreover, it has been recently suggested that one of the functions of p53 is to modulate PKR [37]. In order to determine if PKR activation and apoptosis induction in response to 5-FU depend on p53, we analyzed HCT116 p53^+/+^ and HCT116 p53^−/−^ cell lines. As shown in Figure 5A, PKR and eIF2α were phosphorylated during 5-FU treatment in both cell lines. As index of apoptosis, the cleavage of caspase 3 was produced during 5-FU treatment in both cell lines at 16 and 24 hours post-treatment, and was detected slightly during treatment with IFNα. However, the level of the cleavage of caspase 3 detected during 5-FU treatment was lower in HCT116 p53^−/−^ cells compared to the HCT116 p53^+/+^ cells, further supporting the known involvement of p53 in the apoptosis induced by 5-FU [7]. In addition, PKR total level was also up-regulated in the absence of p53 protein. As expected, PKR was up-regulated in response to IFNα in both cell lines. In order to determine whether up-regulation of PKR is at the transcriptional level, the PKR mRNA was measured by real time PCR in HCT116 p53^+/+^ and p53^−/−^ cells. Whereas IFNα treatment produced about 4-fold induction in both cell lines, 5-FU treatment did not induce significant up-regulation of PKR mRNA compared to untreated cells (Figure 5B). As it has been previously shown, p53 is involved in the apoptosis induced by 5-FU as demonstrated by comparing the apoptosis detected in HCT116 p53^+/+^ and that observed in p53^−/−^ cells. However, down-regulation of the PKR levels by shRNA interference was able to induce a reduction of the apoptosis induced by 5-FU in both cells lines (Figure 5C). Moreover we examined by immunofluorescence in HCT116 p53^+/+^ and HCT116 p53^−/−^ cells the level and localization of phospho- PKR (p-T451) and total-PKR (Figure 6). In both cell lines, the level of phospho -PKR increased during 5-FU treatment and was localized in the cytoplasmic fraction and along the cytoplasmic membrane. Total-PKR also increased during 5-FU treatment in both cell lines and was located in the nucleus and in the cytoplasm (Figure 6A and B). These results suggest the relevance of PKR activation in 5-FU-apoptosis induction even in the absence of p53.

## Discussion

Increased interest has been focused on the identification of new clinical markers with regard to chemotherapeutic response and prognosis in cancer. Since alterations in the mediators of 5-FU-induced apoptosis may account for chemoresistance, the identification of new targets involved in the 5-FU-induced apoptosis is of significant clinical interest. Recently, it has been shown that PKR plays an important role in the induction of apoptosis by doxorubicin and etoposide [37], [38].

In this study we have investigated the role of PKR in the cellular response to 5-FU. Our results demonstrated that PKR was markedly up-regulated and activated during the 5-FU treatment in MEFs and in human colon and breast cancer cell lines, triggering eIF2α phosphorylation (Figure 1). Significantly, after PKR activation there was a marked reduction in PKR phosphorylation levels at 24 h post-treatment (Figure 1A). This effect could be due to phosphatase PP-1 regulation [39] and/or by proteolytic degradation of active PKR mediated by caspase activation during the apoptosis induction [40], [41].

We have found that the colon T84 cancer cell line and the breast T47D cancer cell line did not express PKR protein even in the presence of IFNα (Figure 1B). Curiously, Jurkat cells express a truncated version of PKR with decreased activity [18]. Aberrant activation of PKR has been reported in numerous neurodegenerative diseases and cancer [17], [18], [19], [20]. Loss of expression of PKR has recently been found in immunohistochemical analysis of tumor samples from lung [42]. However, if a truncated version of PKR or the absence of PKR protein expression also occurs in other solid tumors has not yet been analyzed.

Continued activation of PKR finally triggers cell cycle arrest and/or the apoptosis phenomena [43], [44]. The cell cycle arrest induced by 5-FU in MEFs PKR^+/+^ was not detected in absence of PKR protein (Figure 2A). Similar results have been reported for heparin-treatment of PKR^−/−^ MEFs which were markedly insensitive to its antiproliferative actions [45]. In fact, PKR acts as a potent negative regulator of cell growth by the attenuation of cell cycle progression when is over-expressed in yeast or mammalian cells [44], [46]. As shown here, PKR induces apoptosis in response to 5-FU treatment since PKR knockdown decreased significantly this cell death phenomenon (Figure 2B, C and D). It is well established that eIF2α phosphorylation correlates with a translational block and consequently leads to inhibition of protein synthesis, providing the cell with the opportunity to elicit adaptive responses [40], [43]. However, although prolonged induction of eIF2α phosphorylation is mainly proapoptotic [43], [47], transient induction of eIF2α phosphorylation functions mostly cytoprotectively through the activation of pathways that promote cell survival such as the phosphatidylinositol-3 kinase (PI3K) and nuclear factor kB (NF-kB) pathways [48], [49]. Using MEFs expressing a non-phosphorylatable form of eIF2α (eIF2α^A/A^ MEFs), we found during 5-FU treatment higher levels of apoptosis than control cells. This result suggests that similarly as it has been described for doxorubicin treatment [50], the PKR- mediated eIF2α phosphorylation induced by 5-FU would have a protective role prior to the induction of apoptosis. PKR is involved in numerous pathways activated during cellular stress. Although the direct substrate of PKR is the translation initiation factor eIF2α, PKR has wide substrate specificities and might also act through protein-protein interactions [16], [51]. In fact, this kinase acts as a signal integrator for stress-activated protein kinase pathways, leading to stimulation of JNK, p38 and ATF-3 and the induction of apoptosis in response to various stimuli such as dsRNA, LPS, TNF-α and doxorubicin [38], [52], [53], [54], [55].

In correlation with the different levels of apoptosis and PKR expression, the cytotoxic effect produced by 5-FU in cells with PKR knockdown was significantly lower that in cells expressing PKR (Figure 3 and Table 1), suggesting the implication of PKR activation in response to 5-FU treatment. Moreover, several studies showed the effectiveness *in vitro* and *in vivo* of combined therapy with IFNα and 5-FU [56], [57], [58]. Various mechanisms have been reported to be involved in the synergistic effect of 5-FU/IFNα combination therapy, such as reduced 5-FU clearance, and increased amount of FdUMP metabolite together with increased inhibition of thymidylate synthase [57], [59]. Furthermore, analysis in hepatocarcinoma cell lines revealed the importance of apoptosis in the improvement of antitumor activity of 5-FU by IFNα. More recent studies involved p27Kip1, Fas/FasL and TRAIL as mediators of the improvement in apoptosis during drug combination [58], [60], [61]. Our results suggest that the up-regulation of PKR induced by IFNα treatment could be also involved in the effectiveness of combined therapy (Figure 3, Table 1). Cells with PKR knockdown treated with IFNα/5-FU were less affected than cells expressing PKR, as shown by the cytotoxic analysis and levels of apoptosis detected by the combined 5-FU/IFNα treatment (Figure 3, Table 1 and Figure 4). Therefore, our results suggest that PKR protein could be included into the group of mediators of 5-FU action, and that the antitumor activity produced by 5-FU should be further enhanced when PKR is induced by IFNα.

The p53 tumor suppressor has been reported as an important protein involved in 5-FU-induced apoptosis [3], [7]. However, experiments showed that apoptosis can also occur in mutant p53 cell lines by a mechanism still unknown [6]. We have shown here that PKR is activated in absence of p53 expression and PKR knockdown decreased 5-FU- mediated apoptosis in HCT116 p53^+/+^ cells and almost prevented apoptosis in HCT116 p53^−/−^ cells. (Figure 5). These results suggest the importance that both p53 and PKR play in the 5-FU-induced apoptosis, and the relevance acquired by PKR in tumor cells where p53 is mutated. Since the association of p53 tumor alterations with patient prognosis and response to adjuvant chemotherapy has been widely studied, with contradictory findings [62], [63], [64], the analysis of additional prognostic and preventive molecular markers such as PKR could be of clinical interest.

The recent description that p53 could act directly on the PKR promoter in response to genotoxic damage [37] led us to examine whether the up-regulation of PKR protein levels detected in response to 5-FU treatment (Figure 5 and Figure 6) is due to a transcriptional regulation mediated by p53. Our results showed that the increase in PKR protein level in response to 5-FU treatment was partially regulated by p53 since level of PKR increased in absence of p53, although to a lesser extent (Figure 5A and Figure 6). The mRNA analysis suggested that PKR protein up-regulation was not due to a transcriptional phenomenon (Figure 5C). Therefore, similar to what has been described for the molecular targets of 5-FU, thymidylate synthase protein, and even the p53 protein, PKR might undergo a post-transcriptional regulation during 5-FU treatment [65], [66], [67]. Although PKR promotes the proteosomal degradation of p53 through the glycogen synthase kinase 3 [68], if PKR stability protein is also regulated by p53 is still unknown.

To summarize, our results demonstrate that PKR is a 5-FU molecular target that plays an important role in the cytotoxic effect of 5-FU at least, in part, through induction of cell death by apoptosis in a p53 -independent manner. These results suggest the clinical importance that the PKR status could play in response to chemotherapy based on 5-FU. Moreover, the effectiveness of 5-FU cytotoxic activity induced by IFNα, especially in cancer cells expressing a mutated form or lacking p53, but with a functional PKR, might have relevant clinical application in patients.
